# Inulin supplementation ameliorates hyperuricemia and modulates gut microbiota in *Uox*-knockout mice

**DOI:** 10.1007/s00394-020-02414-x

**Published:** 2020-10-26

**Authors:** Yingjie Guo, Yanan Yu, Hailong Li, Xueli Ding, Xiaoyu Li, Xue Jing, Jianwei Chen, Guilin Liu, Yuan Lin, Chen Jiang, Zhen Liu, Yuwei He, Changgui Li, Zibin Tian

**Affiliations:** 1 Department of Gastroenterology, The Affiliated Hospital of Qingdao University, Qingdao, 266000 Shandong China; 2 Institute of Metabolic Diseases, Qingdao University, Qingdao, 266003 Shandong China; 3 BGI-Qingdao, BGI-Shenzhen, Qingdao, 266555 Shandong China; 4 Synthetic Biology Technology Innovation Center of Shandong Province, Qingdao, 266101 Shandong China

**Keywords:** Inulin;, Hyperuricemia, Intestinal barrier, Inflammation, Gut microbiota, Short chain fatty acids

## Abstract

**Purpose:**

Inulin is a type of fermentable dietary fiber, which is non-digestible, and can improve metabolic function by modulating intestinal microbiota. This study aimed to evaluate the role of inulin in hyperuricemia and microbial composition of the gut microbiota in a mouse model of hyperuricemia established through knockout of *Uox* (urate oxidase) gene.

**Methods:**

KO (*Uox*-knockout) and WT (wild-type) mice were given inulin or saline by gavage for 7 weeks. The effect of inulin to combat hyperuricemia was determined by assessing the changes in serum UA (uric acid) levels, inflammatory parameters, epithelial barrier integrity, fecal microbiota alterations, and SCFA (short-chain fatty acid) concentrations in KO mice.

**Results:**

Inulin supplementation can effectively alleviate hyperuricemia, increase the expressions of ABCG2 in intestine, and downregulate expression and activity of hepatic XOD (xanthine oxidase) in KO mice. It was revealed that the levels of inflammatory cytokines and the LPS (lipopolysaccharide) were remarkably higher in the KO group than those in the WT group, indicating systemic inflammation of hyperuricemic mice, but inulin treatment ameliorated inflammation in KO mice. Besides, inulin treatment repaired the intestinal epithelial barrier as evidenced by increased levels of intestinal TJ (tight junction) proteins [ZO-1 (zonula occludens-1) and occluding] in KO mice. Moreover, serum levels of uremic toxins, including IS (indoxyl sulfate) and PCS (*p*-cresol sulfate), were reduced in inulin-treated KO mice. Further investigation unveiled that inulin supplementation enhanced microbial diversity and raised the relative abundance of beneficial bacteria, involving SCFAs-producing bacteria (e.g., *Akkermansia* and *Ruminococcus*). Additionally, inulin treatment increased the production of gut microbiota-derived SCFAs (acetate, propionate and butyrate concentrations) in KO mice, which was positively correlated with the effectiveness of hyperuricemia relief.

**Conclusions:**

Our findings showed that inulin may be a promising therapeutic candidate for the treatment of hyperuricemia. Moreover, alleviation of hyperuricemia by inulin supplementation was, at least, partially conciliated by modulation of gut microbiota and its metabolites.

**Electronic supplementary material:**

The online version of this article (10.1007/s00394-020-02414-x) contains supplementary material, which is available to authorized users.

## Introduction

As a common metabolic disease, hyperuricemia is resulted from the dysregulation of purine metabolism and is featured by increased serum UA (uric acid) levels [1]. The incidence of hyperuricemia has been remarkably increased worldwide, and it has emerged as a public health challenge [2]. From 2006 to 2014, the prevalence of hyperuricemia rose from 19.7 to 25.0% in males and from 20.5 to 24.1% in females in the Irish health system [3]. Besides, it was recently reported that the incidence of hyperuricemia among Chinese females and males was 7.9 and 19.4%, respectively [4]. The number of confirmed cases has reached 170 million by the end of 2017 in China. Hyperuricemia is often accompanied by chronic low-grade inflammation and associated with gout, hypertension, chronic kidney disease, and metabolic alterations, such as hyperlipidemia and diabetes [5–7]. Additionally, recent studies further revealed that hyperuricemia could also affect intestinal microbial homeostasis and gut epithelial integrity [8, 9].

Besides kidneys, the intestine is also involved in UA excretion, and approximately 30% of UA is cleared via intestine [10]. The optimal physiological environment of the intestinal microbiota may be changed by the increased soluble serum UA. In humans, gut microbiota composition differs between hyperuricemic patients and healthy individuals. This altered microbiota is termed dysbiosis, and in comparison with the microbiota of healthy individuals, the abundance of *Bacteroides caccae* is enriched, and *Bifidobacteria* and *Faecalibacteria* are depleted in patients with hyperuricemia [11]. Animal studies have reported some certain beneficial bacteria, including SCFAs (short-chain fatty acids)-producing bacteria (e.g., *Clostridium* and *Ruminococcaceae*), which are decreased in hyperuricemic animal models [8, 12]. Emerging literature suggested that gut dysbiosis not only leaded to bacteria or bacterial products translocation such as LPS (lipopolysaccharide), but also resulted in increased intestinal permeability in hyperuricemia [8]. Probiotics manipulate intestinal microbiota and possess a variety of benefits for gut health, thereby improving UA metabolic outcomes. For instance, lactobacillus brevis DM9218 can attenuate hyperuricemia through modifying gut microbiota disturbance and mucosal barrier function in fructose-induced hyperuricemic mice [13]. Additionally, our previous study also demonstrated the beneficial effect of probiotics, such as *Bifidobacteria* and *Lactobacilli* on hyperuricemia in mice [14]. Therefore, the anti-hyperuricemic effects by modulation of intestinal homeostasis and gut microbiota are well worthy of attention and deep research.

Inulin is a fermentable dietary fiber and is closely dependent on the microbiota in gut since it could not be directly decomposed or absorbed in digestive tract [15]. Inulin acts as an effective prebiotic by stimulating the beneficial bacterial growth, such as *Bifidobacteria* and *Lactobacilli* [16]. Nowadays, inulin is the only prebiotic, which has been approved by the European Food Safety Authority due to its ability in improving the bowel function [17]. Moreover, it has been widely reported that some beneficial bacteria (e.g., *Akkermansia* and *Ruminococcus*) could enzymatically hydrolyze inulin, so as to produce SCFAs in the colon and thus mitigate low-grade inflammatory response and facilitate glucose metabolism [18, 19]. However, whether inulin is able to alleviate hyperuricemia by the modulation of gut microbiota is still unknown and needs to be discovered. The present study aimed to investigate the role of inulin in changing gut microbiota and intestinal barrier in a hyperuricemic mouse model and assess whether such changes could improve UA metabolism. For this purpose, the composition of gut microbiota was detected by using a metagenomic sequencing method, and the levels of UA, SCFAs, inflammatory cytokines, and intestinal barrier function were measured.

## Materials and methods

### Animals and treatment

A KO (Uox-knockout) mouse model for hyperuricemia was generated on a pure C57BL/6J genetic background using the TALEN (transcription activator-like effector nuclease) technology. The mouse model is characterized by stably elevated serum UA, which could be used to investigate hyperuricemia and its related disorders, imitating the human condition [20]. Male WT (wild-type) mice and KO mice with C57BL/6J background, aged 7 weeks, were obtained from Institute of Metabolic Diseases of Qingdao University (Qingdao, China). Animals were kept in the climate- and light-controlled chamber at 23 ± 1 ℃ under a 12/12 h dark/light cycle and given sterile water and standard chow. Table S1 showed components of the standard chow, and the nutrient requirements were achieved for the mice. The KO mice were randomly assigned to two groups, KO group and KO + I group (*n* = 8 for each group) together with 8 WT mice as control. In the KO + I group, mice were daily given inulin by oral gavage (9.5 g/kg/day). In the KO group, mice were given the same amount of saline. Inulin was provided by Sigma-Aldrich Co. Ltd. (St. Louis, MO, USA). The dose selection was dependent on previous studies, in which the selected dose should not exceed the limit of inulin for nutrition supplementation [21, 22].

The body weight of each mouse was measured weekly. After 7 weeks, fresh feces were obtained and frozen in a – 80 °C refrigerator for further microbiota analysis. After fasting for 12 h, mice were sacrificed via CO_2_ inhalation, and the blood samples were collected. Then, the blood samples were centrifuged to obtain serum at 3000 rpm for 10 min. The liver and small intestine were carefully separated and weighed. Serum and all tissues were snap-frozen and kept in a – 80 °C refrigerator until the next experiment. All experimental procedures were approved by the Ethical Committee for Animal Experimentation of The Affiliated Hospital of Qingdao University (approval number: AHQU20150109).

### Analysis of biochemical parameters

From the beginning of the experiments, the serum UA levels of each mouse were measured weekly by an automatic biochemical analyzer (Toshiba, Tokyo, Japan). Blood of the mice was drawn from the tail vein at each time point. The serum levels of LPS, DAO (diamine oxidase), d-LAC (d-lactate), IS (indoxyl sulfate), and PCS (*p*-cresol sulfate) were detected using commercial ELISA (enzyme-linked immunosorbent assay) kits (Cusabio Technology, Wuhan, China; Future industrial, Shanghai, China). The activity of hepatic XOD (xanthine oxidase) was determined using ELISA kits (Beijing Solarbio Science & Technology Co., Ltd., Beijing, China).

Intestine tissues were homogenized by using mortar and pestle with PBS (phosphate-buffered saline) (pH 7.4). The supernatant of the homogenates was obtained for further measurement of inflammatory cytokines following centrifugation for 20 min at 3000 rpm. The levels of inflammatory cytokines [IL-1β (interleukin-1β), TNF-α (tumor necrosis factor-alpha), and IL-6 (interleukin-6)] in the intestinal tissue and serum were detected by using ELISA kits (ABclonal Biotechnology Co., Ltd., Wuhan, China). All the experiments were performed according to manufacturers’ instructions.

### Quantitative reverse transcription polymerase chain reaction (qRT-PCR)

Total RNA in small intestine and liver tissues was extracted by applying TRIzol regent (Tiangen Biotech Co., Ltd., Beijing, China). EasyScript Plus cDNA synthesis kit (Takara Bio, Shiga, Japan) was used to synthesize cDNA according to the manufacturer’s protocol. qRT-PCR assay was carried out in a reaction system of 25 µl by applying TB Green Premix Ex Taq reagent (Takara Bio, Shiga, Japan) with the fluorescent PCR instrument (CFX96; Bio-Rad Laboratories Inc., Hercules, CA, USA). The amplification reaction was undertaken as follows: at 95 °C for half a minute, followed by 40 cycles at 95 °C for 5 s and at 60 °C for half a minute. Besides, β-actin, as a house-keeping gene, was used as a reference. Relative gene expression levels were determined using the 2$^{{ - \Delta \Delta {\text{C}}_{{\text{t}}} }}$ method. The primer sequences for the target genes are listed in Table S2.

### Western blot analysis

Proteins were extracted from the intestine tissue samples by using RIPA (radioimmunoprecipitation assay) buffer (Beyotime Institute of Biotechnology, Shanghai, China). Then, the concentrations of proteins were detected by the BCA (bicinchoninic acid) assay kit (Thermo Fisher Scientific, Inc., Waltham, MA, USA). Moreover, 10% SDS-PAGE (sodium dodecyl sulfate-polyacrylamide gel electrophoresis) was used for separation of 30 μg protein from each sample. Afterwards, the proteins were transferred onto 0.45 µm PVDF (polyvinylidene fluoride) membranes (Millipore, Billerica, MA, USA). Following blocking by skimmed milk (5% w/v), the primary antibodies [ZO‑1 (1:1000; 5406, Cell Signaling Technology, USA), occludin (1:1000; A2601; ABclonal Biotech, Wuhan, China) and GAPDH (1:1000; 8884; Cell Signaling Technology, USA)] were incubated together with the membranes overnight in a 4 °C refrigerator. Then, secondary antibodies were used for incubation. An ECL (enhanced chemiluminescence) reagent was added to PVDF membranes, so that the protein bands could be visualized. Image J software (provided by National Institutes of Health, Bethesda, MD, USA) was applied to calculate the intensity of each protein band. The level of GAPDH (glyceraldehyde 3-phosphate dehydrogenase) was set as the internal control.

### Metagenome sequencing and analysis

Total DNA was obtained from the fecal samples by utilizing the QIAamp DNA Stool Mini Kit (provided by Qiagen, Hilden, Germany), in accordance with the manufacturer’s instructions. First, the extracted DNAs were split into 300–700 base pairs (bps) by applying AMPure XP beads (Beckman Coulter Inc., Brea, CA, USA) and Covaris E220 (Covaris Inc., Woburn, MA, USA). The DNA fragments between 200–400 bp were selected and used for the metagenomic library construction using the MGIEasy DNA Rapid Library Prep Kit (BGI, catalog no, 1000006985) following the manufacturer’s instructions. And the circularization metagenomic DNB (DNA nanoball)-based libraries were generated. Afterwards, BGISEQ-500 platform (MGI Tech Co., Ltd, Shenzhen, China) was used in the sequencing of the libraries in a model of paired ends to acquire a read of 100 bp in length, as described previously [23]. After filtered the low quality, duplication and adapter contamination reads, the clean data of each sample was assembled into contigs using the idba (v1.1.3) software. After gene prediction by MetaGenemark (v3.6.2), the non-redundant gene set was generated using the CD-Hit (v4.6.3) software and the gene abundance profile was generated using SOAP-aligner(v2.22). Alpha-diversity, including community richness index (Chao1) and community diversity index (Shannon), and PCoA (Principal component analysis) were analyzed with the QIIME software (v1.9). The linear discriminant analysis (LDA) effect size was used to evaluate the differences in bacterial taxa among the groups by LEfSe software (*P* < 0.05 and LDA score > 2.5). The gene set function was annotated by KEGG (Kyoto Encyclopedia of Genes and Genomes) database (v87.0) using diamond (v0.8.23.85) with *E*-value < 1e-5, and then combined with the gene set abundance to generate the metabolic function profile of gut microbiota [24]. The pathway significant comparison was calculated using wilcox test and visualized by STAMP (v2.1.3). Correlation analysis of UA levels, fecal SCFAs concentration, differential microorganisms, intestinal barrier and inflammation markers were performed by “spearman” method using R (v3.4.1). The data reported in this study are available in the CNGB Nucleotide Sequence Archive (CNSA: https://db.cngb.org/cnsa; accession number CNP0000964).

### Analysis of fecal SCFAs

Analysis of fecal SCFAs was carried out as reported previously [25]. In brief, feces were homogenized with distilled water and H_2_SO_4_ solution. Following acidification, SCFAs were extracted by using diethyl ether. The extracted SCFAs were centrifuged for 20 min at 12,000 rpm to acquire the supernatant. The levels of SCFAs, which included acetate, propionate and butyrate, were detected with the GC–MS (gas chromatography–mass spectrometry) using an ISQ LT GC–MS system (Thermo Fisher Scientific, Inc., Waltham, MA, USA) accompanied by the WAX capillary column (30 m × 0.25 mm × 0.25 μm). Helium (1.0 ml/min) was used as the carrier gas. The temperatures for ionization and injection were 200 and 180 ℃, respectively.

### Statistical analysis

Results were presented as mean ± SEM (standard error of the mean). All statistical analyses were performed using SPSS 19.0 (IBM Inc., Armonk, NY, USA) and Graph Prism 6.0 (GraphPad Software, Inc., San Diego, CA, USA) software. ANOVA (one-way analysis of variance) followed by Tukey’s post hoc test was utilized to compare differences between the groups. *P* < 0.05 was considered statistically significant.

## Results

### Effects of inulin on serum levels of UA in KO mice

**Fig. 1:**
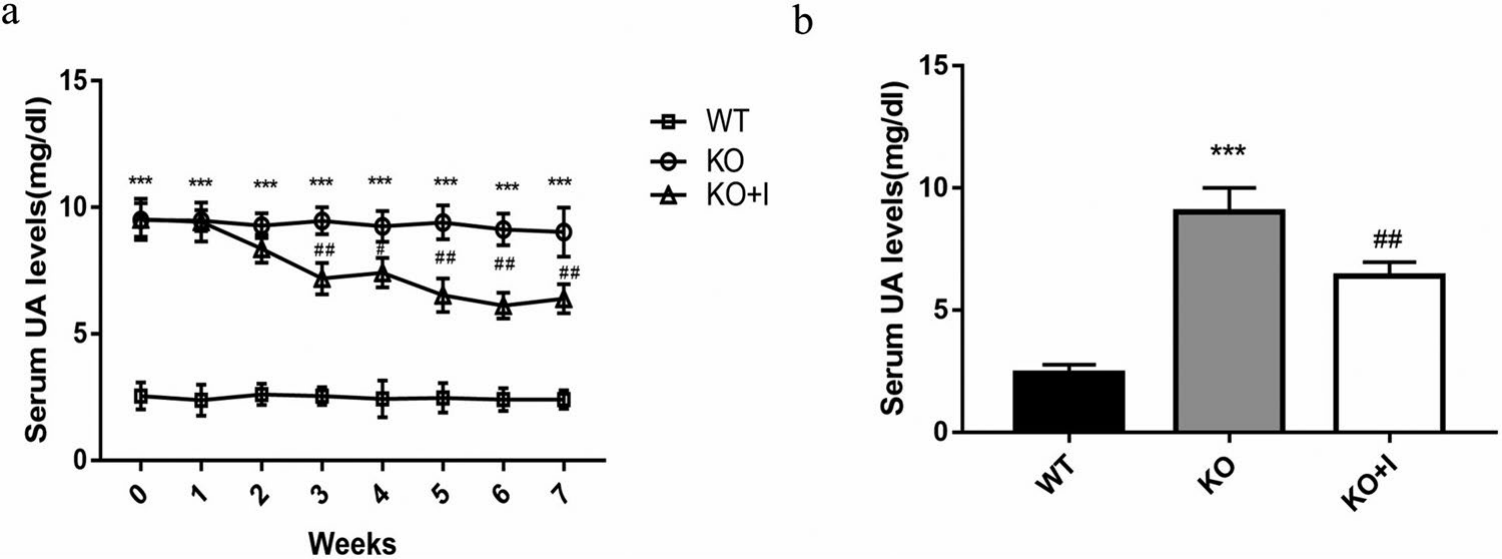
Effects of inulin on serum UA levels in KO mice. **a**, **b** Serum UA levels were detected for 7 weeks. Data are shown as mean ± SD (*n* = 8). *P < 0.05, **P < 0.01, ***P < 0.001 versus WT group; ^#^*P* < 0.05, ^##^*P* < 0.01, ^###^*P* < 0.001 versus the KO group

There was no significant difference in the baseline body weight of mice in the three groups. The changes of body weight in the three groups were similar, without significant difference during the 7 weeks dietary intervention (Table S3). The level of UA in the KO group (9.51 ± 0.46 mg/dl) was remarkably increased compared with that in the WT group (2.55 ± 0.33 mg/dl), indicating that the hyperuricemic mouse model was successfully established. Inulin interventions caused a significant reduction in the serum level of UA from the end of the third week onward in KO + I group (*P* < 0.01) (Fig. 1a). As shown in Fig. 1b, the serum level of UA in KO + I group was reduced by 30% after 7 weeks inulin intervention (6.39 ± 0.20 mg/dl) compared with that in the KO group, while that was still above a normal level in the WT group.

### Effects of inulin on the mucosal barrier function in KO mice

**Fig. 2:**
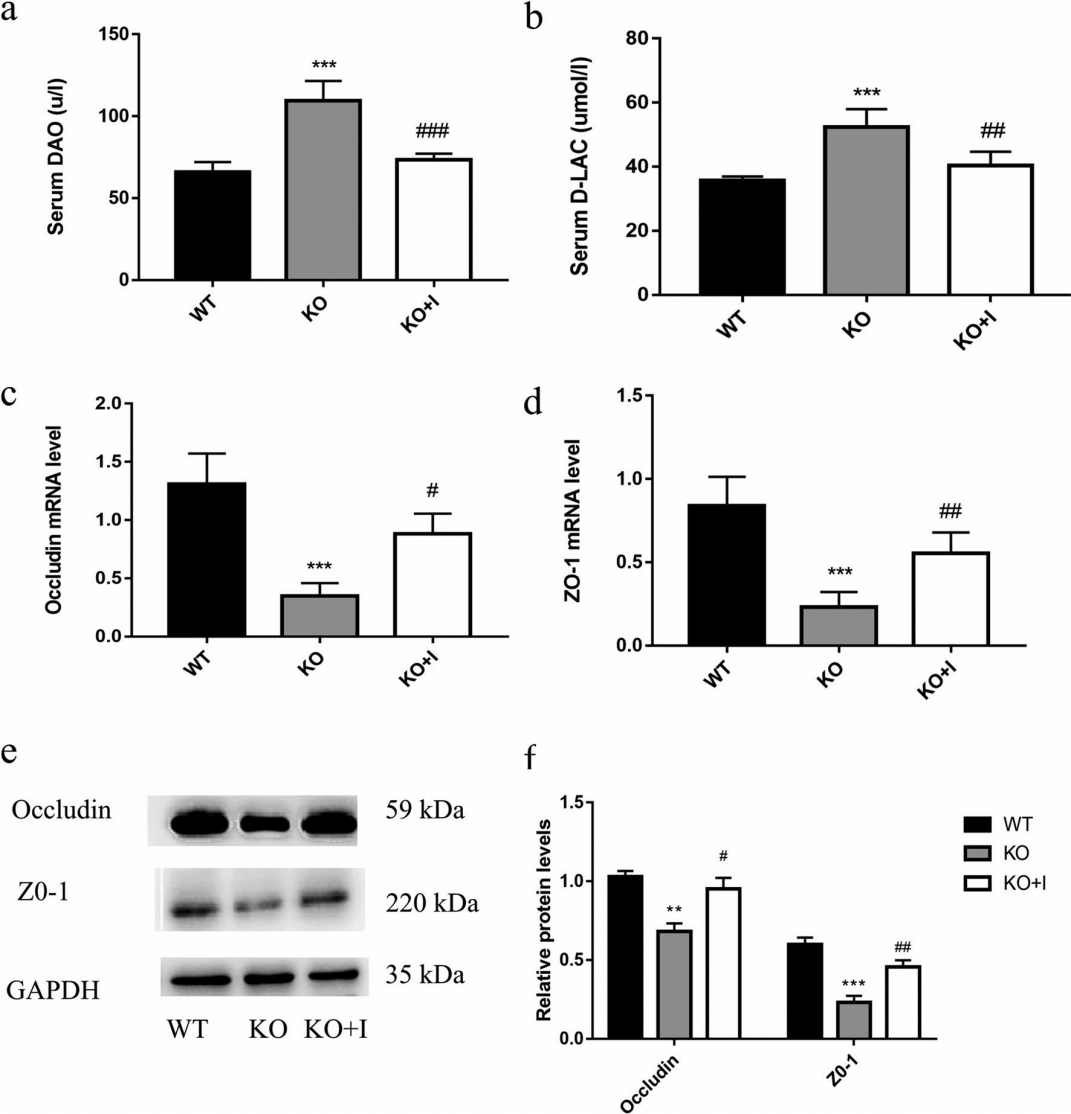
Effects of inulin on the intestinal mucosal barrier in KO mice. **a** Serum DAO, **b** Serum d-LAC, **c**, **d** The mRNA levels of occludin and ZO-1, **e**, **f** The protein levels of occludin and ZO-1. Data are shown as mean ± SD (*n* = 8). *P < 0.05, **P < 0.01, ***P < 0.001 versus WT group; ^#^*P* < 0.05, ^##^*P* < 0.01, ^###^*P* < 0.001 versus the KO group

Serum levels of d-LAC and DAO were measured. As illustrated in Fig. 2a, b, the WT mice had the lowest serum levels of DAO and d-LAC, and the KO mice had the highest serum levels of d-LAC and DAO. Inulin treatment could reduce the serum levels of DAO and d-LAC compared with those in the KO group. To further evaluate the mucosal barrier function in intestine, the protein and mRNA levels of tight junction proteins in intestinal tissues of mice were detected by using Western blotting and qRT-PCR. The data showed that the expression levels of occludin and ZO-1 in KO mice were lower than those in WT mice. However, after dietary inulin supplementation, the levels of these proteins were markedly increased in KO + I group in comparison with those in KO group (Fig. 2c–f). The above-mentioned results demonstrated that inulin could promote the repair of intestinal barrier in KO mice.

### Effects of inulin on the levels of systemic endotoxin and inflammatory cytokines in KO mice

**Fig. 3:**
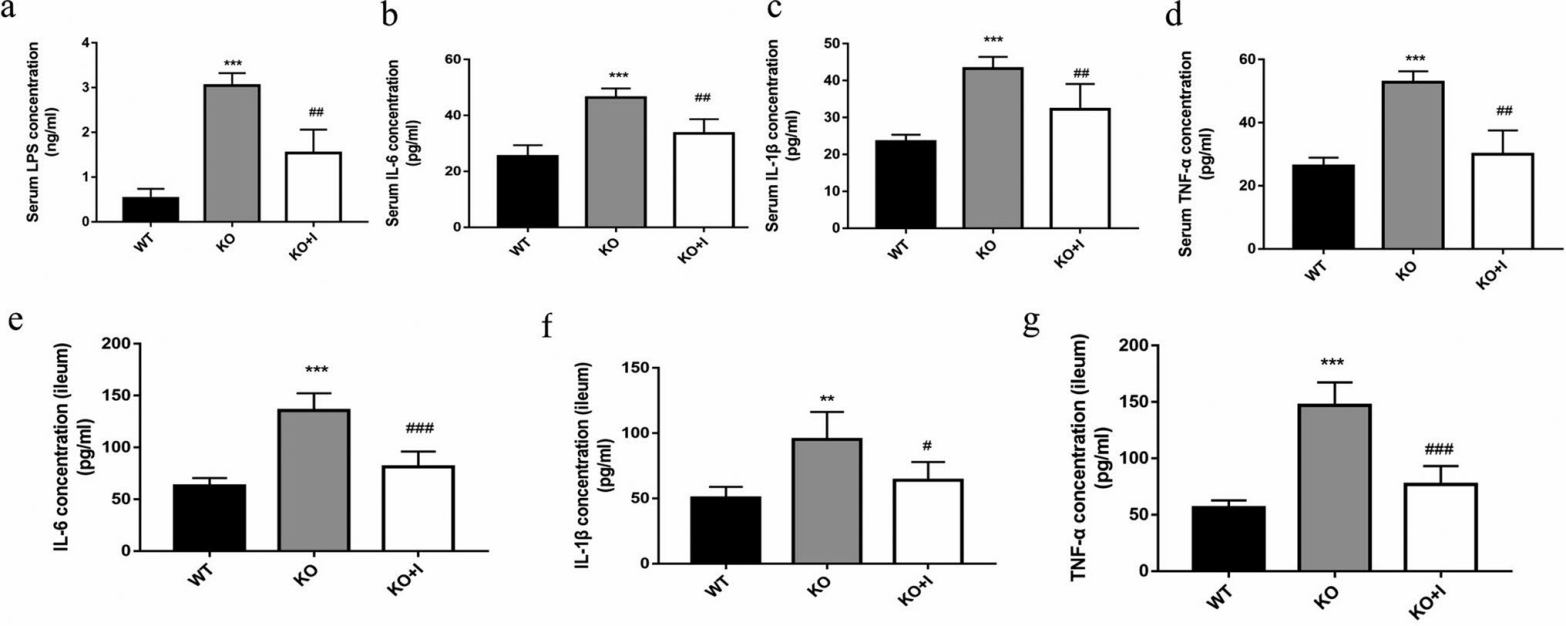
Effects of inulin on systemic endotoxemia and inflammation of KO mice. The serum levels of lipopolysaccharide (LPS) (**a**), IL-6 (**b**), IL-1β (**c**), TNF-α (**d**), and IL-6 (**e**), IL-1β (**f**), TNF-α (**g**) concentrations in intestinal tissues. Data are shown as mean ± SD (*n* = 8). *P < 0.05, **P < 0.01, ***P < 0.001 versus WT group; ^#^*P* < 0.05, ^##^*P* < 0.01, ^###^*P* < 0.001 versus the KO group

Previous studies indicated that individuals with hyperuricemia suffer from a low-grade inflammation systemically [5]. In order to explore the functions of inulin in inflammation and endotoxemia in KO mice, the levels of LPS and inflammatory cytokines, such as TNF-α, IL-6 and IL-1β, were detected. As depicted in Fig. 3, the serum levels of LPS in the KO group were increased in comparison with those in the WT group (*P* < 0.001). Furthermore, the levels of IL-1β, IL-6 and TNF-α in intestine and serum were remarkably higher in the KO group compared with those in the WT group. It was revealed that inulin treatment reduced the LPS level and the concentrations of inflammatory cytokines in the KO group, suggesting that inulin could attenuate the levels of systemic endotoxin and inflammatory cytokines in hyperuricemic mice.

### Effects of inulin on hepatic XOD and intestinal UA transporters in KO mice

**Fig. 4:**
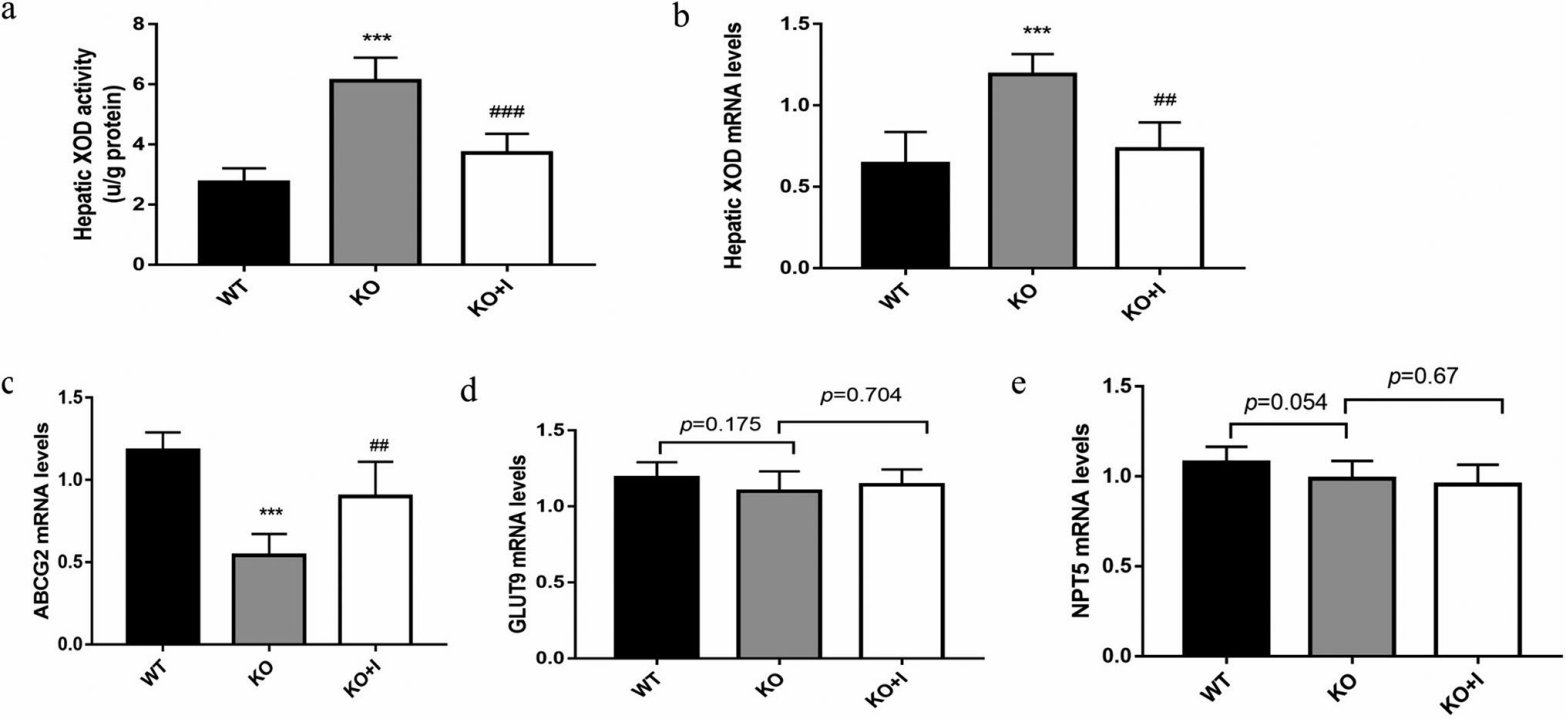
Effects of inulin on hepatic XOD and intestinal UA transporters in KO mice. Hepatic XOD activity (**a**), the mRNA levels of XOD (**b**), ABCG2 (**c**), GLUT9 (**d**) and NPT5 (**e**). Data are shown as mean ± SD (*n* = 8). *P < 0.05, **P < 0.01, ***P < 0.001 versus WT group; ^#^*P* < 0.05, ^##^*P* < 0.01, ^###^*P* < 0.001 versus the KO group

XOD is one of the core enzymes in liver, converting hypoxanthine and xanthine to UA [26]. As displayed in Fig. 4a, b, hepatic XOD activity and mRNA levels were remarkably higher in the KO group than those in the WT group (both, *P* < 0.001). Nonetheless, inulin could notably decrease hepatic XOD activity and suppress XOD expressions in the KO + I group. These findings indicated that XOD could be normalized by inulin supplementation, resulting in a reduction in UA production.

Several urate transporters that contribute to UA secretion have been identified in the intestines, such as ABCG2 (ATP-binding cassette transporter G2), GLUT9 (glucose transporter 9), and NPT5 (NPT homolog). In the present study, the relative gene expression level of ABCG2 was markedly decreased in the KO group compared with that in the WT group, while inulin treatment significantly increased ABCG2 expression in the KO + I group. However, the expressions of GLUT9 and NPT5 in intestinal tissues showed no significant difference among the three groups (Fig. 4c–e).

### Effects of inulin on the SCFAs levels in KO mice

**Fig. 5:**
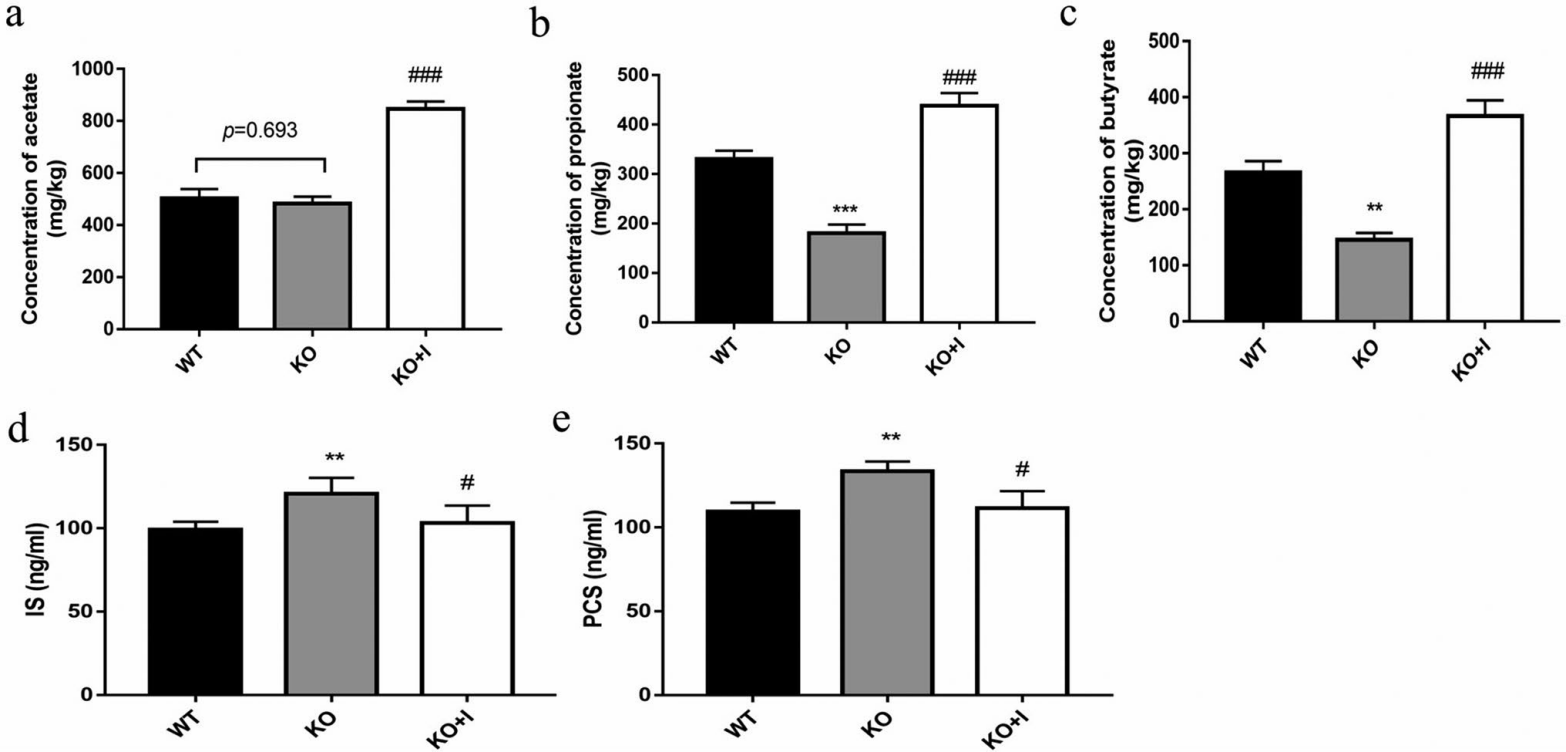
Effects of inulin supplementation on the SCFAs levels and uremic toxins in KO mice. Acetate (**a**), propionate (**b**) and butyrate (**c**) concentration in fecal samples, the serum levels of indoxyl sulfate (**e**) and *p*-cresol sulfate (**f**). Data are shown as mean ± SD (*n* = 8). *P < 0.05, **P < 0.01, ***P < 0.001 versus WT group; ^#^*P* < 0.05, ^##^*P* < 0.01, ^###^*P* < 0.001 versus the KO group

SCFAs are the terminal products of colonic microbial fermentation, which are signaling molecules between the host and gut microbiota and indicative for host health. We detected the levels and types of SCFAs existing in feces from WT and KO mice with or without inulin supplement. In the KO group, the levels of the main fecal SCFAs, such as propionate and butyrate, were decreased compared with those in the WT group, while no significant difference was noted in acetate levels between the WT group and KO group. The levels of fecal acetate, propionate and butyrate were all increased after inulin treatment in the KO + I group compared with those in the KO and WT groups (all, *P* < 0.001). The above-mentioned findings indicated that inulin could increase production of SCFAs (Fig. 5a–c).

### Effects of inulin on the serum levels of uremic toxins in KO mice

IS (indoxyl sulfate) and PCS (*p*-cresol sulfate) are unique co-metabolites produced by gut microbiota and representative uremic toxins. As displayed in Fig. 5d, e, the serum concentrations of IS and PCS were higher in the KO group compared with those in the WT group. After treatment with inulin, the levels of IS and PCS were markedly attenuated in the KO + I group (both, *P* < 0.05).

### Influences of inulin on gut microbiota in KO mice

**Fig. 6:**
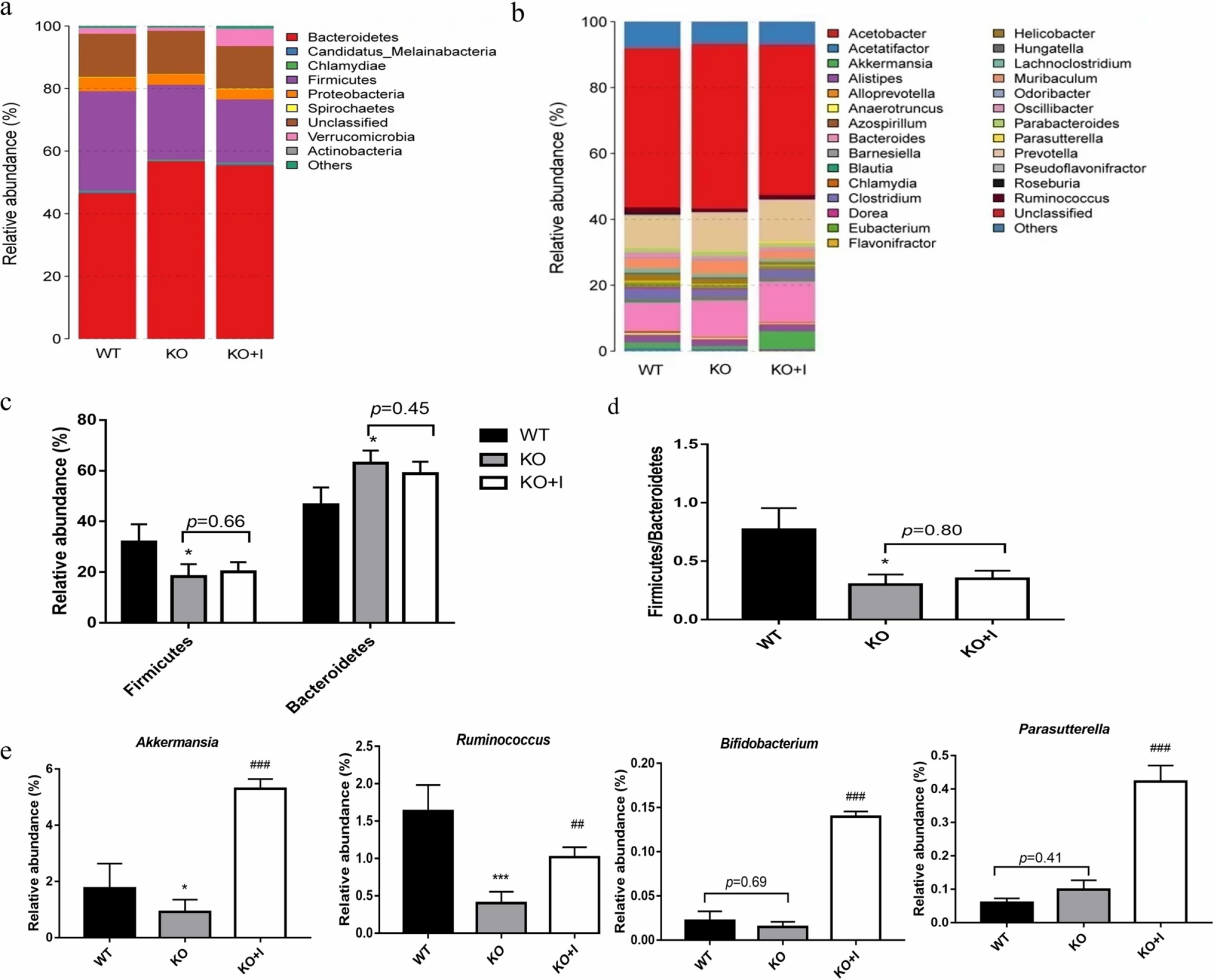
Inulin supplementation modulated the composition of the gut microbiota. **a** Microbial composition at the phylum level, **b** microbial composition at the genus level, **c** relative abundance of Firmicutes and Bacteroidetes at the phylum level, **d** Firmicutes/Bacteroidetes (F/B) ratio, **e** the relative abundance of *Akkermansia*, *Ruminococcus*, *Bifidobacterium* and *Parasutterella.* Data are shown as mean ± SD (*n* = 8). *P < 0.05, **P < 0.01, ***P < 0.001 versus WT group; ^#^*P* < 0.05, ^##^*P* < 0.01, ^###^*P* < 0.001 versus the KO group

The molecular ecological analysis of fecal microbiota was performed by metagenomic sequencing. To identify specific taxa related to inulin supplementation, the relative abundance of the bacterial profile was assessed at the phylum and genus level. The mice from the KO group showed remarkable shifts in gut microbial composition and structure versus the control group (Fig. 6a, b). In line with the previous research, the Bacteroidetes and Firmicutes were found mainly predominant in composition of gut microbiota in mice [27]. Compared with the WT group, the relative abundance of Firmicutes reduced, while Bacteroides increased in the KO group (Fig. 6c). The KO group had a significantly lower Firmicutes/Bacteroidetes ratio compared with the WT group, which was not reversed by inulin supplementation (Fig. 6d). However, inulin administration increased the relative number of Verrucomicrobia in the KO + I group. At the genus level, a relative higher number of *Bacteroides* was found in the KO group, while a relatively lower number of *Akkermansia* and *Ruminococcus* was noted in comparison with that in the WT group. Conversely, the lowered abundance of *Akkermansia* and *Ruminococcus* was significantly elevated by inulin treatment in the KO mice. Meanwhile, inulin administration increased the relative abundance of *Parasutterella* and *Bifidobacterium* in the KO + I group (Fig. 6e).

**Fig. 7:**
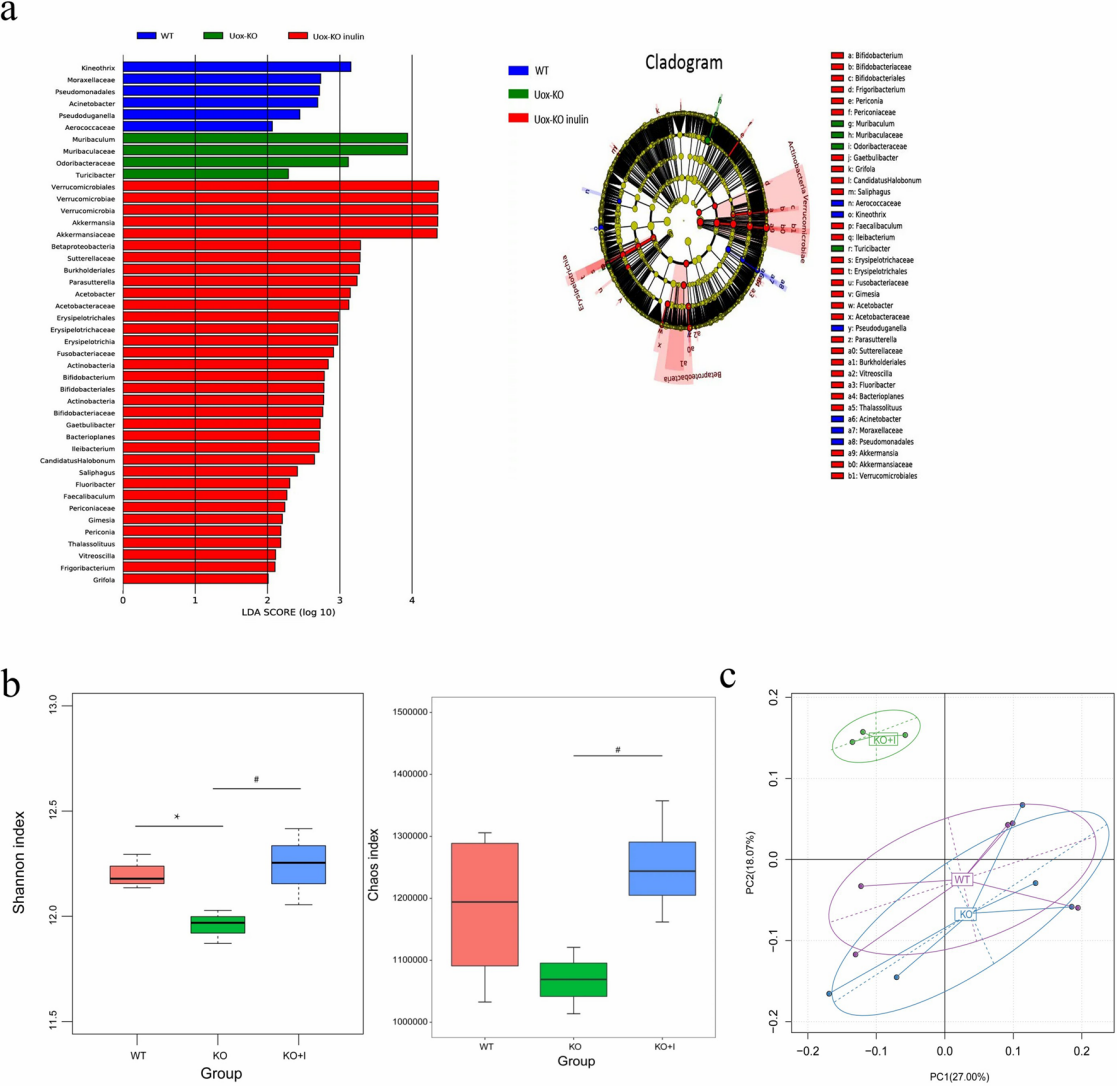
LEfSe comparison and diversity analysis of microbiota in all groups. **a** LEfSe comparison of microbiota in all groups (histogram of LDA scores and taxonomic cladogram), **b** α diversity indicated by Chao1 and Shannon indices, **c** β diversity based on principal coordinates analysis (PCoA)

To further explore the changes in the key microbiota among the three groups, the LEfSe analysis was applied. Using histogram of LDA scores and taxonomic cladogram, biomarkers were authenticated and the dominant microorganisms were shown in the groups. As illustrated in Fig. 7a, Kineothrix, Moraxellaceae, Pseudomonadales, *Acinetobacter* were the main microorganisms in WT group; Muribaculaceae and *Odoribacteraceae* were the main microorganisms in KO group; while *Akkermansia*, *Parasutterella*, *Bifidobacterium* were the dominant microbes in KO + I group. Additionally, the Shannon and Chao1 diversity indices were evaluated to indicate gut microbial diversity (Fig. 7b). The Shannon index was remarkably lower in the KO group than that in the WT group (*P* < 0.05). Treatment with inulin increased the Shannon and Chao1 indices versus the KO group. PCoA (principal coordinates analysis) of weighted UniFrac distance reflected Beta diversity, showing differences in gut microbial composition among all groups (Fig. 7c).

**Fig. 8:**
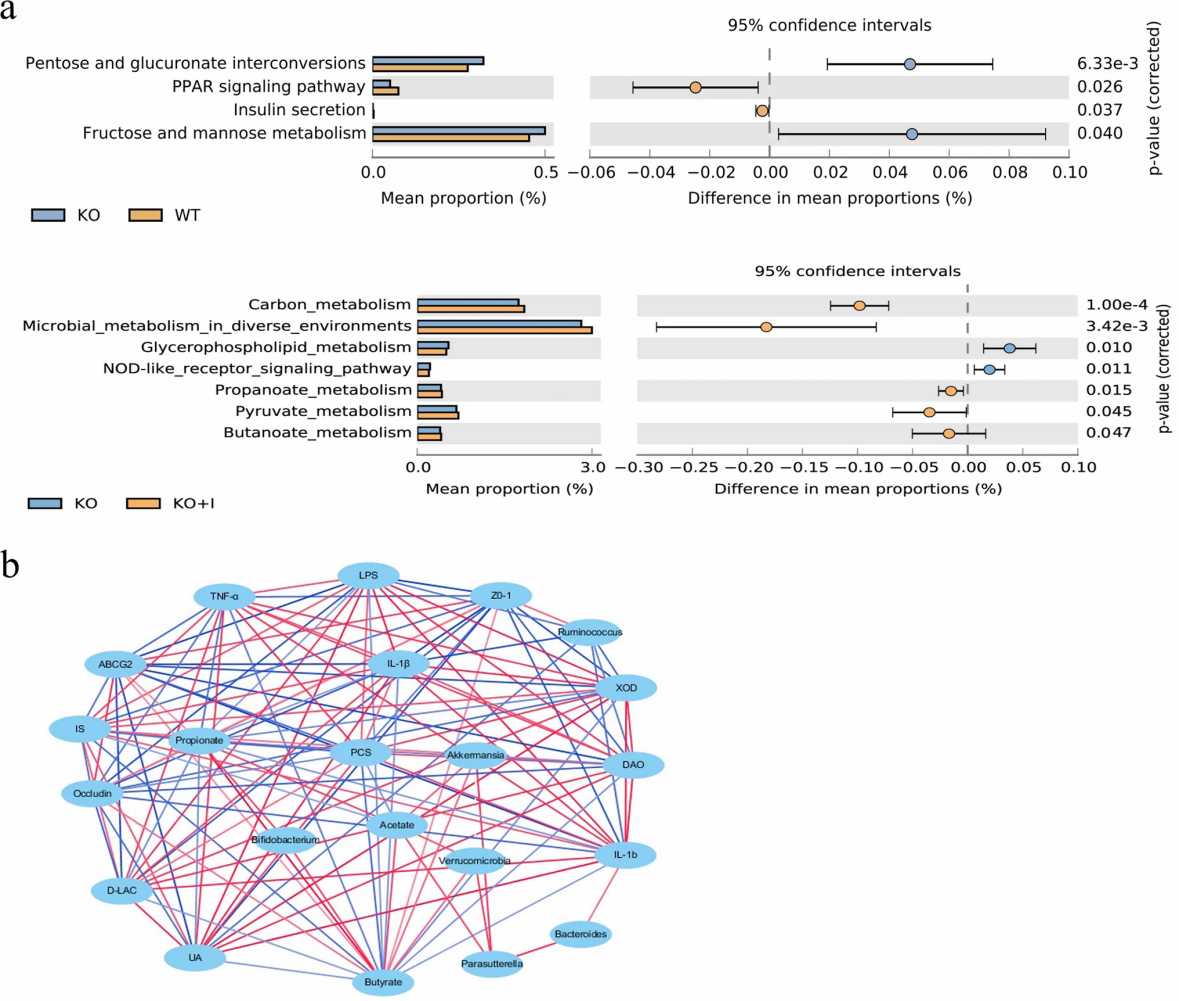
Functional prediction of altered gut microbiota and correlation networks. **a** differential KEGG pathways between the three groups, **b** the interaction networks among UA levels, SCFAs production, differential microorganisms, intestinal barrier and inflammation markers. The edge colors indicated positive (red) or negative (blue) correlations (*P* < 0.05, *r* > 0.4 or *r* < − 0.4)

The potential metabolic functions of the gut microbiota would be caused by alterations in bacterial taxa. The gut microbiota functions were analyzed based on the KEGG database. As shown in Fig. 8a, four different functional pathways were identified between the WT and KO mice. Pathways involved in pentose and glucuronate interconversions, and fructose and mannose metabolism were upregulated, whereas other two pathways, e.g., PPAR (peroxisome proliferator-activated receptor) signaling pathway and insulin secretion pathway, were downregulated in the KO mice. Inulin treatment upregulated butanoate metabolism, propanoate metabolism, and microbial metabolism in diverse environments (Fig. 8a).

Furthermore, the correlations among UA levels, fecal SCFAs concentration, differential microorganisms, intestinal barrier and inflammation markers were analyzed. Figure 8b showed that UA levels were positively related to inflammatory cytokines (TNF-α, IL-1β and IL-6), LPS, and XOD, but negatively related to TJ proteins (ZO-1and occludin), propionate, butyrate, and *Ruminococcus* (*P* < 0.05, *r* > 0.4 or *r* < − 0.4). Obviously, hyperuricemia was closely associated with intestinal barrier, inflammatory cytokines, and gut microbiota. According to network analysis, intestinal barrier and inflammation markers were directly affected by the gut microbiome and SCFAs. *Ruminococcus* positively correlated with TJ proteins (ZO-1), in contrast, negatively correlated with inflammatory cytokines (IL-1β and IL-6), LPS, and UA, indicating *Ruminococcus* had a positively effect on hyperuricemia. However, *Bacteroides* showed positively correlations with IL-1β. Inulin interventions regulated the gut microbiota, and thus changed SCFAs production indirectly. *Akkermansia* was positively correlated with acetate and butyrate, *Parasutterella* was positively correlated with acetate, and *Bifidobacterium* was positively correlated with butyrate and propionate through the network analysis. The increased SCFAs (acetate, propionate and butyrate) were positively related to TJ proteins (ZO-1 and occludin), and negatively correlated with inflammatory cytokines (TNF-α, IL-1β and IL-6), LPS, uremic toxins (IS and PCS) and UA, indicating that SCFAs production may be significant for hyperuricemia relief by inulin treatment (Fig. 8b).

## Discussion

Inulin has been used as a prebiotic to alleviate metabolic disorders, such as obesity and diabetes by modulating the gut microbiota [28–30]. The present study was performed to assess the long-term influences of inulin on experimental hyperuricemic mice and its possible mechanisms. The hyperuricemic mouse model was successfully established to investigate the anti-hyperuricemic effects of inulin. The results of the current study suggested that inulin could improve intestinal barrier function, alleviate inflammatory state, and reduce serum UA levels in the KO mice. Importantly, treatment with inulin enhanced microbial diversity and raised the abundance of specific bacteria, including *Akkermansia* and *Ruminococcus*, which regulated intestinal microorganisms and increased the production of SCFAs. The serum UA level was significantly reduced by 30% in KO mice after 7 weeks inulin intervention, while that was higher than the normal level (Fig. 1b). In our previous study, administration of allopurinol (100 mg/kg) for 2 weeks reduced the serum UA level to a normal level in KO mice [20]. Compared with urate-lowering drugs, anti-hyperuricemic efficacy of inulin seemed limited. The limited effects of inulin supplementation on hyperuricemia may be due to the restoration of gut dysbiosis and intestinal barrier function, as well as suppression of inflammation, potentially providing a theoretical foundation on inulin as an adjuvant treatment for hyperuricemia.

Intestine is an important organ for UA excretion. Endogenous UA from blood is secreted directly into the intestinal lumen at all intestinal segments via urate transporter [10, 31]. The excretion of UA via intestine depends on the homeostasis of the gut that is maintained by intestinal mucosal barrier [32]. Intestinal mucosa intact inter-cellular junctions form the basis for segregation of microbes and endotoxin, thereby preventing inflammation-related conditions in the intestine [33]. In hyperuricemic animal models, both the fructose-induced hyperuricemic mice and the KO mice have increased intestinal permeability and impaired intestinal barrier in comparison with their non-hyperuricemic controls [8, 9, 13]. In the present study, we found decreased expressions of tight junction proteins (occludin and ZO-1) and increased intestinal permeability in the KO mice, accompanied by increased serum levels of endotoxin, DAO, and d-lactate. Inulin treatment prevented hyperuricemia-induced barrier damage via enhancing the expression levels of occludin and ZO-1 in the intestinal epithelium (Fig. 2). ABCG2, as a high-capacity urate transporter expressed on the apical membrane in intestine, plays a vital role in UA excretion. It has been reported that the expression level of ABCG2 was significantly decreased during inflammation and negatively correlated with impaired intestinal barrier [34]. In the present research, the mRNA level of ABCG2 was lower in the KO group than that in the WT group. Additionally, inulin supplementation led to a significant increase in intestinal expression of ABCG2 in KO mice (Fig. 4c). Our results supported an effective role of inulin in maintenance of intestinal epithelial barrier of integrity and intestinal UA excretion in hyperuricemic mice.

The results of the present study uncovered that the levels of inflammatory cytokines (IL-6, IL-1β, and TNF-α) and the LPS in the serum and intestinal mucosa were remarkably higher in the KO group than those in the WT group, indicating that hyperuricemic mice were in a low-grade systemic inflammation. This inflammatory response is likely due to the increased intestinal permeability and gut dysbiosis observed in KO mice with hyperuricemia [8, 9, 35]. Inulin supplementation can effectively relieve the inflammatory reaction in KO mice. It was reported that inflammatory cytokines and LPS could upregulate the expression and activity of hepatic XOD [13, 36, 37]. Our results revealed that the activity of hepatic XOD was positively correlated with inflammatory cytokines (TNF-α, IL-1β and IL-6) and LPS (Fig. 8b). The KO mice exhibited a higher XOD mRNA level and activity than WT mice in inflammatory responses, which was restored by inulin treatment in the current research (Fig. 4a, b). It was disclosed that the normalization of XOD activity may be related to decrease in inflammatory responses after inulin treatment, resulting in reduction in UA production.

Numerous studies have revealed that gut microbiota is an important factor associated with hyperuricemia [8, 12, 38]. The elevated serum level of UA may change physiological environment of the gut microbiota and contribute to changes in composition and diversity of intestinal microbiota. In agreement with the results of previous studies, a decreased relative abundance of *Firmicutes* and an increased relative abundance of *Bacteroidetes* were observed in KO mice [8]. An elevated ratio of *Bacteroidetes*/*Firmicutes* may be a feature of hyperuricemia-driven disruptions in microbiota, while supplementation with inulin did not prevent these changes (Fig. 6c, d). The microbial diversity has been shown to be reduced in hyperuricemic individuals [11]. The results of the current study showed that the alpha-diversity of fecal microbiota was markedly lower in the KO group than that in the WT group, which highlighted that there was a dysbiotic status in bacterial communities of KO mice [39, 40]. In the present study, the alterations of microbial diversity were restored by inulin treatment, indicating possible protective effects of inulin on gut microbiota (Fig. 7b).

In the current study, there was a raise in opportunistic pathogens *Bacteroides*, and a decreased number of beneficial microbiota *Akkermansia* and *Ruminococcus* was noted in the KO group compared with that in the WT group (Fig. 6). Some *Bacteroides fragilis* were found to invade intestinal tissues and cause damage [41]. *Akkermansia* and *Ruminococcus* are known to be involved in SCFAs production, and their relative abundance has been found to be associated with improved host health [42, 43]. Additionally, *Akkermansia* is a type of probiotic, which represents the phylum *Verrucomicrobia* available in the microbial community of gut [44]. It has been reported that the elevated abundance of *Akkermansia* is associated with improved metabolic profiles, such as glucose intolerance, insulin resistance, steatohepatitis, and chronic inflammation [45, 46]. Our results revealed that *Ruminococcus* were positively correlated with ZO-1 and negatively correlated with IL-1β, IL-6, LPS and UA, while *Bacteroides* were positively correlated with IL-1β. Inulin exhibited no significant influence on the number of *Bacteroides*, whereas a reverse effect was noted on the *Ruminococcus* and *Akkermansia*. In addition, inulin supplementation could remarkably increase the relative abundance of *Bifidobacterium* and *Parasutterella* (Fig. 6). The genus *Bifidobacterium* has been directly utilized as probiotic therapy to relieve hyperuricemia. *Parasutterella* has been reported to be correlated with various health outcomes via improving the metabolism of aromatic amino acid, bilirubin and purine, while the potential biological functions of *Parasutterella* in hyperuricemia need to be further investigated [47]. Thus, the ameliorating effect of inulin on hyperuricemia is closely related to the alteration of gut microbiota.

SCFAs are major bacterial metabolites and affect various physiological processes of the host [48, 49]. SCFAs, for instance, propionate, acetate, and butyrate, are vital metabolites for the maintenance of intestinal homeostasis. In the current study, treatment with inulin increased acetate, propionate and butyrate concentrations versus the KO group (Fig. 5a–c). *Akkermansia* was positively correlated with acetate and butyrate, *Parasutterella* was positively correlated with acetate, and *Bifidobacterium* was positively correlated with butyrate and propionate through the network analysis (Fig. 8b). KEEG analysis demonstrated that pathways involved in metabolism of SCFAs, such as microbial metabolism in diverse environments, and metabolism of butanoate and propanoate, were upregulated by inulin treatment, and it might be associated with the increased levels of propionate and butyrate (Fig. 8a). SCFAs, especially propionate and butyrate, were reported to provide ATP to the cells of the intestinal wall to exhibit beneficial effects on UA excretion [50, 51]. A study reported that rectal administration of butyrate may be efficacious in improving metabolism of UA in healthy volunteers [52]. Our results revealed the increased propionate and butyrate were positively related to TJ proteins (ZO-1 and occludin), and negatively correlated with inflammatory cytokines (TNF-α, IL-1β and IL-6), LPS and UA, indicating that SCFAs production may be involved in hyperuricemia relief by inulin treatment. The uremic toxins (IS and PCS) are unique co-metabolites produced by gut microbiota, which are associated with CKD (chronic kidney disease) progression [53]. Intestinal dysbiosis may contribute to the increase in IS and PCS [54]. Peng et al. indicated that the serum levels of IS and PCS were increased in a hyperuricemic rat model induced by potassium oxonate [55]. In the present study, the serum levels of IS and PCS in the KO group were significantly higher compared with those in the WT group, while inulin supplementation decreased the levels of uremic toxins (Fig. 5d, e). The elevated levels of uremic toxins in hyperuricemic mice could be partly attributed to the dysbiotic gut microbiota and impaired intestinal barrier. Moreover, the results showed that the levels of IS and PCS displayed highly negative correlation with acetate, propionate and butyrate. We speculated that inulin ameliorating hyperuricemia in KO mice may be partly attributed to modulation of gut microbiota, as well as influencing production of gut microbiota-derived SCFAs.

In summary, we found that inulin could improve gut barrier, ameliorate inflammation, and decrease UA levels in serum in KO mice. Inulin increased the number of beneficial bacteria and the production of SCFAs as well. The alleviation of hyperuricemia by inulin supplementation was, at least, partially conciliated by modulation of gut microbiota and its metabolites. However, whether the positive therapeutic effects of inulin are mediated by changes in the gut microbiota should be further explored via fecal microbiota transplantation. Our findings may provide a key evidence that inulin may become a promising therapeutic candidate for treating hyperuricemia and the associated disorder.

## Electronic supplementary material

Below is the link to the electronic supplementary material.

Supplementary file1 (DOC 54 kb)
