# Supplementary material for: Inulin supplementation ameliorates hyperuricemia and modulates gut microbiota in Uox-knockout mice
Source: Eur J Nutr. 2020 Oct 26;60(4):2217–30. doi: 10.1007/s00394-020-02414-x (PMC8137640; doi:10.1007/s00394-020-02414-x)
Supplement: Supplementary file 1 — Supplementary file1 (DOC 54 kb) [file 394_2020_2414_MOESM1_ESM.doc]

**Table S1 The component of standard laboratory chow.**

| Component | Content (g/kg) |
| --- | --- |
| Water | 95.0 |
| Crude protein | 220 |
| Crude fat | 50 |
| Crude fiber | 30 |
| Crude ash | 60 |
| Calcium | 12.5 |
| Phosphorus | 7.5 |
| Lysine | 15.9 |
| Methionine+Cystine | 9.9 |

Table S2 Primer sequence.

| Gene name | Direction | Primer sequences |
| --- | --- | --- |
| ZO-1 | Forward | 5′-CCACCTCTGTCCAGCTCTTC-3′ |
|  | Reverse | 5′-CACCGGAGTGATGGTTTTCT-3′ |
| Occludin | Forward | 5'-CCTCCAATGGCAAAGTGAAT-3' |
|  | Reverse | 5'-CTCCCCACCTGTCGTGTAGT-3' |
| XOD | Forward | 5'-ATGACGAGGACAACGGTAGAT-3' |
|  | Reverse | 5'-TCATACTTGGAGATCATCACGGT-3' |
| ABCG2 | Forward | 5′-GAACTCCAGAGCCGTTAGGAC-3′ |
|  | Reverse | 5′-CAGAATAGCATTAAGGCCAGGTT-3′ |
| GLUT9 | Forward | 5′-TTGCTTTAGCTTCCCTGATGTG-3′ |
|  | Reverse | 5′-GAGAGGTTGTACCCGTAGAGG-3′ |
| NPT5 | Forward | 5′-AGCAAACCTCCGAGATAG-3′ |
|  | Reverse | 5′-CTGGCAAAGACAGAAGAC-3′ |
| β-actin | Forward | 5'-CTCCCTGGAGAAGAGCTATGA-3' |
|  | Reverse | 5'-GGCATAGAGGTCTTTACGGATG-3' |

**Table S3 The body weight of the three groups during the seven-week dietary intervention.**

| Weeks | WT | KO | KO+I | *P* |
| --- | --- | --- | --- | --- |
| 0 | 22.01±0.38 | 21.95±0.41 | 21.82±0.29 | 0.71 |
| 1 | 23.01±0.17 | 22.88±0.21 | 22.96±0.26 | 0.63 |
| 2 | 24.64±0.32 | 24.30±0.36 | 24.59±0.22 | 0.61 |
| 3 | 25.17±0.79 | 25.05±0.61 | 25.32±0.93 | 0.59 |
| 4 | 26.35±0.62 | 26.14±0.58 | 26.17±0.67 | 0.89 |
| 5 | 27.52±0.47 | 27.31±0.56 | 27.35±0.53 | 0.64 |
| 6 | 28.26±0.63 | 28.15±0.66 | 28.29±0.71 | 0.77 |
| 7 | 29.21±0.29 | 29.08±0.35 | 29.13±0.46 | 0.42 |

Values represented as means ± standard deviations (n= 8). The changes of body weight in the three groups were similar, without significant difference during the seven-week dietary intervention.
